# Effect of *Lycium barbarum* Polysaccharide on Decreasing Serum Amyloid A3 Expression through Inhibiting NF-*κ*B Activation in a Mouse Model of Diabetic Nephropathy

**DOI:** 10.1155/2022/7847135

**Published:** 2022-01-29

**Authors:** Fengqi Wan, Fulin Ma, Jiaxin Wu, Xinyu Qiao, Minxue Chen, Wenjian Li, Liang Ma

**Affiliations:** ^1^Institute of Modern Physics, Chinese Academy of Sciences, No. 509 Nanchang Road, Lanzhou 730000, China; ^2^University of Chinese Academy of Sciences, No. 19A Yuquan Road, Beijing 100049, China; ^3^Cuiying Biomedical Research Center, Lanzhou University Second Hospital, No. 82 Cuiyingmen, Lanzhou 730030, China; ^4^School of Pharmacy, Lanzhou University, No. 99 Donggang West Road, Lanzhou 730000, China

## Abstract

*Lycium barbarum* polysaccharide (LBP) as one of the main bioactive constituents of the fruit of *Lycium barbarum* L. (*LB*L.) has many pharmacological activities, but its antihyperglycemic activity is not fully understood yet. This study investigated the hypoglycemic and renal protective effects of LBP on high-fat diet/streptozotocin- (HFD/STZ-) induced diabetic nephropathy (DN) in mice. Blood glucose was assessed before and after 8-week administration of LBP, and the homeostasis model assessment-insulin resistance (HOMA-IR) index was calculated for evaluating the antidiabetic effect of LBP. Additionally, serum creatinine (sCr), blood urea nitrogen (BUN), and urine microalbumin were tested to evaluate the renal function. HE and PAS stainings were performed to evaluate the morphology and injury of the kidney. The results showed that LBP significantly reduces the glucose level and ameliorates the insulin resistance of diabetic mice. Importantly, LBP improves renal function by lowering the levels of sCr, BUN, and microalbumin in diabetic mice and relieves the injury in the renal glomeruli and tubules of the DN mice. Furthermore, LBP attenuates renal inflammation as evidenced by downregulating the mRNA levels of *TNFα*, *IL1 β*, *IL6*, and *SAA3* in the renal cortex, as well as reducing the elevated circulating level and protein depositions of SAA3 in the kidney. In addition, our western blot results showed that NF-*κ*B p65 nuclear translocation and the degradation of inhibitory *κ*B-*α* (I*κ*B*α*) occurred during the progress of inflammation, and such activated signaling was restrained by LBP. In conclusion, our findings suggest that LBP is a potential antidiabetic agent, which ameliorates the inflammation in DN through inhibiting NF-*κ*B activation.

## 1. Introduction

Diabetic nephropathy (DN) is one of the most common complications of diabetes mellitus, and it develops in approximately 40% of diabetic patients [1]. The established therapeutic strategies may slow the progression of renal damage but provide imperfect protection [2]. Identifying new therapeutic targets and treatments that affect the pathogenesis of diabetic nephropathy is of great clinical importance [3].

There has been increasing enthusiasm for using herbal medicines as therapeutic agents for the treatment of diabetes mellitus and its complication DN [4, 5]. *LB*L., a solanaceous defoliated shrubbery, is widely distributed in arid and semiarid regions of northwestern China [6]. The fruit of *LB*L., called wolfberry, has been documented in several traditional Chinese medicine (TCM) books to treat *Xiaoke Bing* [7, 8]. This syndrome is characterized by excessive drinking, eating, emaciation, polyuria, or turbidity and sweetness of urine; thus, diabetes is considered belonging to the scope of *Xiaoke Bing* by TCM [9, 10]. Several Chinese proprietary medicines (final dosage forms of traditional Chinese medicine) that contain the fruit of *LB*L. have been produced and approved to be used in the treatment of diabetes in China, such as Xiaoke Ling tablet, Xiaoke Ping tablet, Yangyin hypoglycemic tablet, and Tangniaole capsule [11].

*Lycium barbarum* polysaccharide (LBP) is one of the main bioactive constituents of *LB*L., and it has multiple biological activities, such as antioxidant, hypoglycemic, hypolipidemic, and anticancer effects, as well as hepatoprotection, immune-modulation, and neuroprotection [12, 13]. Among them, the hypoglycemic effect of LBP has been widely studied both in research and clinical trials [14–16]. An increasing number of studies have evidenced the capacity of crude or purified LBP extracts to influence the blood glucose homeostasis and the inflammatory status responsible for diabetic complications [17].

The pathogenesis of DN is complex, and inflammation is an important player in the pathophysiology of DN [18–20]. The relationships between inflammation and the progression of diabetic nephropathy involve complex molecular networks [21]. Nuclear factor-*κ*B (NF-*κ*B), a transcription factor, serves as a major regulator of inflammatory factors, leading to the overproduction of cytokines in inflammation [22–24]. It is activated in both experimental models and patients of DN, associating with proteinuria and renal interstitial inflammatory cell infiltration [3, 21].

Serum amyloid A (SAA) is a family of acute phase-reactants that exert numerous proinflammatory actions in many cells and tissues including the kidney [25–27]. In mice, SAA isoform 3 (SAA3) is primarily expressed in the kidney, which is most comparable to the isoforms found in humans [28]. SAA has been considered a potential biomarker and therapeutic target of DN [29].

Here, we show the hypoglycemic and renal protective effects of LBP in diabetic mice. LBP significantly reduces the glucose level and insulin resistance in mice and attenuates renal inflammation by suppressing NF-*κ*B activation and the overexpression of inflammatory cytokines and the potential biomarker of DN, SAA3. Our findings suggest that LBP could be an alternative therapy to prevent DN.

## 2. Materials and Methods

### 2.1. Materials

The fruit of *LB*L. was harvested from the coteau of Jingyuan, Gansu Province, China. Quality control of them was identified by Jiaxin Wu in the School of Pharmacy, Lanzhou University, according to the identification standard of Pharmacopoeia of China [11]. Voucher specimens of the fruit of *LB*L. were deposited at the School of Pharmacy, Lanzhou University, for further reference. LBP was obtained from the fruits of *LB*L. by the method of water extraction and ethanol precipitation in our laboratory. Chemical analysis indicated that LBP was composed of arabinose, galactose, glucose, galacturonic acid, mannose, and rhamnose at a molar ratio of 12.25 : 8.66 : 7.66 : 2.86 : 1.70 : 1.00 (Figure S1) [30].

The following reagents were employed in this study: metformin (Squibb & Merck Serono, China); streptozotocin (Sigma, USA); glucose, sCr, BUN, and urine microalbumin kits (Nanjing Jiancheng, China); mouse insulin ELISA kit (Qiaoyu Biotechnology Co., China); SAA3 ELISA kit (J&l Biological Co., China); total RNA extractor (Trizol) and qRT-PCR kits (Takara, Japan); BCA/Bradford protein quantitation kit, hematoxylin-eosin staining kit, and periodic acid-Schiff staining kit (Solarbio, China); immunohistochemistry kit (MXB Biotechnology, China); rat anti-SAA3 (abcam, USA); rabbit anti-NF-*κ*B p65 and mouse anti-I*κ*B*α* (Cell Signaling Technology, USA); rabbit anti-GAPDH, rabbit anti-Lamin B1, biotin-conjugated goat anti-rat IgG, HRP-conjugated goat anti-rabbit IgG, and HRP-conjugated goat anti-mouse IgG (proteintech, USA); and enhanced chemiluminescence kit (meilunbio, China).

### 2.2. Animals

C57BL/6 male mice (18-23 g) were purchased from Lanzhou University. Animals were acclimatized to the new environment for a week; then, they were fed with a free intake of water and maintenance diets. All the animal protocols were approved by the Animal Research Committee of Lanzhou University Second Hospital (No. D2019-069, Lanzhou, China).

### 2.3. Induction of DN Mouse Model and Treatments

To evaluate the hypoglycemic activity of LBP, the diabetic mouse model was established in a similar way as previously described [5, 19, 31, 32]. Briefly, the mice were randomized into two groups including the control group (fed with normal diet, *n* = 10) and the HFD group (fed with high-fat diet from Research Diets, D12492, the formulation is showed in Table S1). After 3 months of feeding, streptozotocin (STZ; 40, 40, 40, and 40 mg/kg body weight; dissolved in 0.1 M citrate buffer (pH 4.5), final volume 0.1 mL/100 g body weight) was administrated intraperitoneally to HFD mice once every 2 days (8 days in total) to induce the diabetic mouse model. Blood glucose was measured from tail vein blood samples using an ACCU-CHEK Active glucometer (Roche, Hoffmann, Germany). Animals with blood glucose > 11.1 mM were considered diabetic [33–35]. Then, the diabetic mice from the HFD group were subdivided into five groups: diabetic group (fed with HFD and equal amount of normal saline, oral gavage; *n* = 10), MET group (positive group, fed with HFD and metformin, the effective dose of 400 mg/kg body weight assayed by preexperiment (Figure S2), dissolved in saline, oral gavage; *n* = 10), and LBP groups (low: 40, medium: 80, and high: 160 mg/kg body weight, dissolved in saline, oral gavage; *n* = 10 of each group, total 30). The body weight of the control and experimental groups was monitored every one or two weeks during the whole process. After 8 weeks of administration, 24 h urinary samples were collected. Then, all the mice were sacrificed after starvation overnight. Blood and tissue samples of the kidney were collected as follows: Leaving the blood samples undisturbed at room temperature for 30 min, the serum and plasma (heparin anticoagulant tubes) were separated by centrifuging at 3000 rpm for 10 min at 4°C. Urine was centrifuged at 3000 rpm for 20 minutes at 4°C, and the supernatant was collected. Serum, plasma, and urine samples were stored at -80°C until the assays were performed using commercial kits. The longitudinal section of the kidney was fixed in 10% neutral buffered formalin and embedded in paraffin for histopathological examination, and the remaining part was stored in liquid nitrogen for further molecular assay. The procedure is summarized in Figure S3.

### 2.4. Biochemical Indexes

Fasting plasma blood glucose-8 weeks (FBG-8w) was measured by a glucose diagnostic kit, and the fasting plasma insulin level was assayed by an ELISA kit. A homeostasis model assessment-insulin resistance (HOMA-IR) was used to evaluate insulin resistance with the following formulae: HOMA − IR = fasting plasma glucose (mM) × fasting plasma insulin (mU/L)/22.5 [36]. Serum creatinine and BUN, plasma SAA3, and urine microalbumin were measured by commercial kits according to the manufacturer's instructions.

### 2.5. Histopathological Examination of the Renal Tissue

Tissue embedded in wax was cut into 4 *μ*m thick sections, and the sections were put in a 55°C oven heating for 3 h. They were routinely dewaxed to hydrate and stained with hematoxylin and counterstained with eosin using standard histological techniques [37]. For periodic acid-Schiff (PAS) staining, the sections were stained with periodic acid-Schiff using standard histological techniques [38].

### 2.6. Immunohistochemistry of the Kidney Tissue

The immunostaining for mouse SAA3 was followed by the immunohistochemistry (IHC) procedures. The deparaffinized and hydrated tissue sections were subjected to antigen retrieval for 10 minutes at 97°C in citrate buffer (pH 6.0). Kidney sections were incubated overnight at 4°C with anti-SAA3 (1 : 50, cat No. ab231680, abcam). The primary antibody was detected using the goat anti-rat biotinylated antibody and visualized with 3,3′ diaminobenzidine (DAB). Sections were counterstained with hematoxylin, and images were captured on an Olympus microscope (BX53, Japan). SAA3 immunostaining abundance and intensity were assessed by a blinded observer (pathologist). Scoring of glomerular and tubulointerstitial compartments was based on staining of the area (0, 25, 50, 75, and 100%) and intensity (0: none, 1: light, 2: medium, and 3: dark). The immunostaining score was a product of area and intensity scores [29].

### 2.7. Quantitative Real-Time Polymerase Chain Reaction (qRT-PCR)

The total RNA of the kidney cortex was extracted using the Trizol reagent. RNA concentration and quality were assessed using the spectrophotometers (Nanodrop 2000, Thermo Fisher) and 1.5% agarose gel electrophoresis. According to the manufacturer's instruction, reverse transcription was performed on 700 ng of total RNA using the cDNA kit. Primers used are shown in Table 1, and qRT-PCR was performed in the instrument of Rotor-Gene. The thermocycling conditions were as follows: initial denaturation at 95°C for 30 sec, 40 cycles of annealing at 95°C for 10 sec, and extension at 60°C for 30 sec. mRNA levels of all genes were normalized to the internal reference gene *β*-actin according to the 2^-*ΔΔ*C^_T_ method [39].

### 2.8. Western Blotting Assay

Western blotting was performed as previously described [40]. Kidneys were homogenized in radioimmunoprecipitation assay buffer (RIPA) and then centrifuged at 12,000 rpm at 4°C to extract the proteins. The nuclear and cytoplasmic proteins were separated using the Active Motif's Nuclear and Cytoplasm Extraction kit according to the manufacturer's protocol. A total of 50 *μ*g protein/lane was loaded onto 12% SDS-PAGE and then transferred to polyvinylidene fluoride membranes. The membranes were then blocked for 1 h at room temperature with 5% skim milk and incubated with anti-NF-*κ*B p65 (1 : 1000, cat. No.8242T, CST), anti-I*κ*B*α* (1 : 1000, cat No. 4814T, CST), anti-Lamin B1 (1 : 1000, cat No. 12987-1-AP, proteintech), and anti-GAPDH (1 : 10000, cat. No. 10494-1-AP, proteintech) overnight at 4°C. After washing with Tris-buffered saline containing Tween 20 (TBST), membranes were incubated with horseradish peroxidase-conjugated secondary antibodies (1 : 5000) for 1 h at room temperature. The immunoreactivity in protein bands was visualized via enhanced chemiluminescence, and the intensity of the bands was measured by ImageJ software. Each experiment was performed at least in triplicate.

### 2.9. Statistical Analysis

All the data were expressed as the mean ± standard deviation (SD). Statistical significance was determined by one-way analysis of variance (one-way ANOVA), and post hoc Tukey's or Dunnett's multiple comparisons test was used. All data were analyzed with GraphPad Prism (Version 7), and the differences were considered significant at *p* < 0.05.

## 3. Results

### 3.1. LBP Decreased Blood Glucose Levels of Diabetic Mice

**Figure 1:**
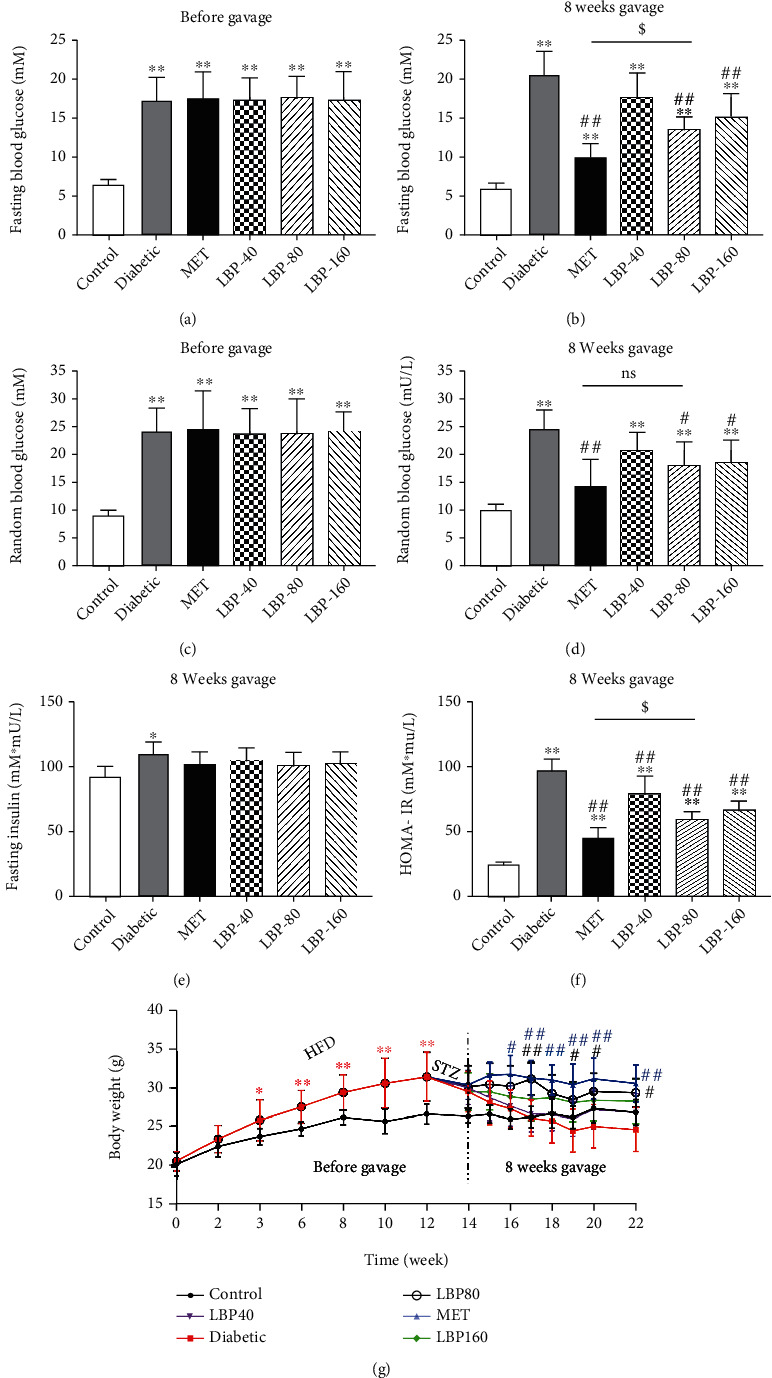
Effects of LBP on blood glucose and insulin resistance of diabetic mice: (a) fasting glucose level before gavage, (b) fasting glucose level after 8 weeks of treatment, (c) random glucose level before gavage, (d) random glucose level after 8 weeks of treatment, (e) fasting insulin level after 8 weeks of treatment; (f) homeostasis model assessment-insulin resistance (HOMA-IR) index after 8 weeks of treatment; (g) body weight monitoring. Data represent the mean ± SD (*n* = 6-9 mice per group). ^∗^Control vs. other groups, ^∗^*p* < 0.05,  ^∗∗^*p* < 0.01; ^#^diabetic vs. other groups, ^#^*p* < 0.05,  ^##^*p* < 0.01; ^$^MET vs. LBP-80, ^$^*p* < 0.05,  ^$$^*p* < 0.01; ns means no significance.

The diabetic mouse model was successfully established with a fasting blood glucose level (FBG) of 11.4~23.8 mM (mean, 17.4 ± 3.0 mM) and random blood glucose level (RG) of 13.3~33.3 mM (mean, 24.1 ± 4.8 mM) after streptozotocin injection (Figures 1(a) and 1(c)). It revealed that the 8-week administration of LBP modulated the blood glucose level significantly and the medium concentration (80 mg/kg body weight) showed the best effect, and the levels of FBG and RG decreased significantly compared with the diabetic group (FBG: 34.1%, RG: 27.3% reductions). Compared to the MET group (FBG: 51.7%, RG: 42.6% reductions), there was no significant difference in the random blood glucose level but a significant difference in the fasting blood glucose level of the LBP group (*p* < 0.05, Figures 1(b) and 1(d)). Additionally, only the level of insulin in the diabetic group (1.2-fold) was significantly higher than in the control group, which was eliminated by LBP and MET treatment (Figure 1(e)). All three doses of LBP significantly reduced the HOMA-IR indexes compared to the diabetic group (19.2%, 39.3%, and 31.9% reductions, respectively, *p* < 0.01), while MET showed the strongest effect on relieving insulin resistance (54.1% reduction, *p* < 0.01, Figure 1(f)). The high-fat diet caused a significant increase in body weight during the modeling period in a time-dependent manner (0-12 weeks), and it was obviously decreased under STZ-induced diabetic conditions. The medium concentration LBP, as well as MET administration, significantly slowed down weight loss during the 8 weeks of gavage compared with the diabetic mice (Figure 1(g)). Collectively, these data indicated that LBP treatment significantly improves glucose metabolism disorder by decreasing blood glucose and alleviating insulin resistance. Among the individual doses of LBP groups, the medium one was found to be the best effective dosage and used in further experiments to evaluate its renal protective effect.

### 3.2. Effects of LBP on Renal Dysfunction of Diabetic Mice

**Figure 2:**
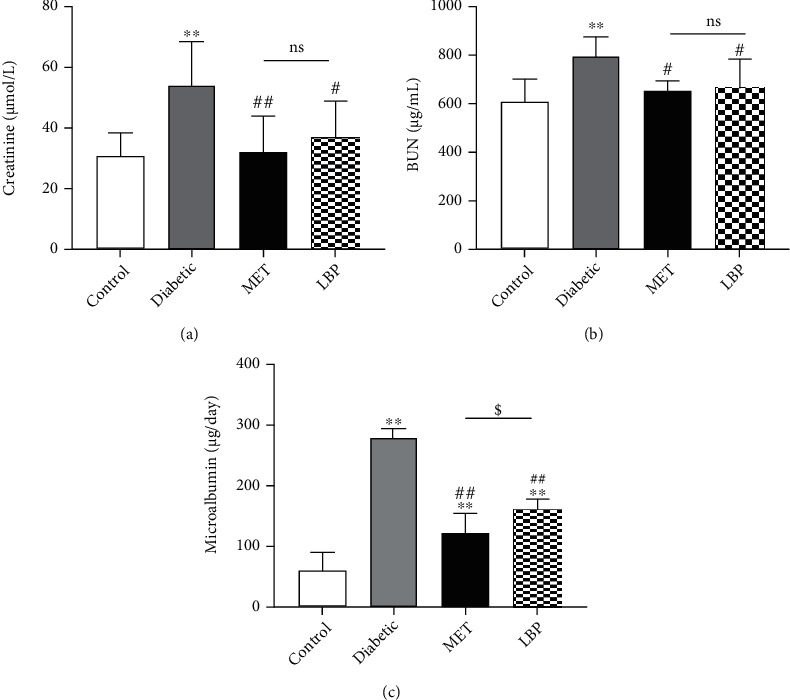
Effects of LBP treatment on creatinine, blood urea nitrogen, and microalbumin in diabetic mice: (a) creatinine in serum; (b) urea nitrogen in serum; (c) microalbumin (24 h) in urine. Data are expressed as the mean ± SD (*n* = 6-9 mice per group). ^∗^Control vs. other groups, ^∗^*p* < 0.05,  ^∗∗^*p* < 0.01; ^#^diabetic vs. other groups, ^#^*p* < 0.05,  ^##^*p* < 0.01; ^$^MET vs. LBP, ^$^*p* < 0.05,  ^$$^*p* < 0.01; ns means no significance.

Serum creatinine (sCr) and blood urea nitrogen (BUN) are important factors of renal dysfunction, and their levels can reflect the damage degree of the glomerular filtration functions [32]. They were increased remarkably by 1.7- and 1.3-fold, respectively, in the diabetic group compared to those in the control group (*p* < 0.01). However, treatment with LBP and MET caused a significant reversal in these biochemical parameters (sCr: 32.0%, BUN: 16.1% reductions for LBP treatment; sCr: 40.6%, BUN: 17.9% reductions for MET treatment; Figures 2(a) and 2(b)). Albuminuria, considered the hallmark for diabetic nephropathy [31, 41, 42], strikingly increased by 4.6-fold in diabetic mice compared to the control group. A significant decrease of the microalbumin level was observed after 8 weeks of LBP and MET treatments in diabetic mice (41.7%, 57.1% reductions, respectively; Figure 2(c)).

### 3.3. Effects of LBP on Renal Histopathological Changes in Diabetic Mice

**Figure 3:**
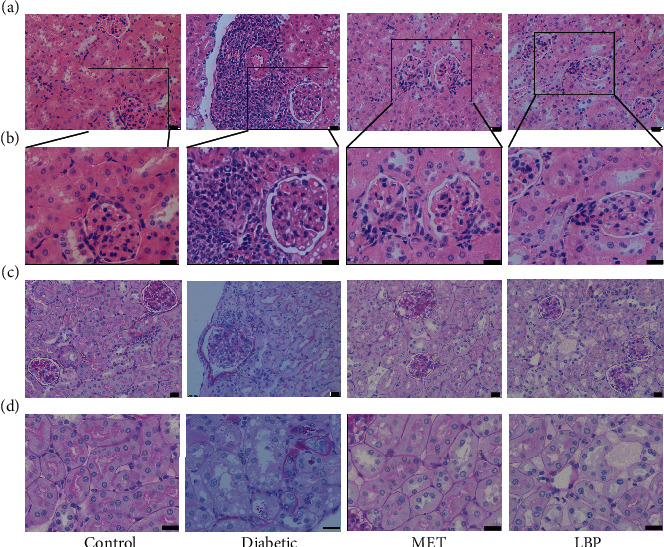
Histopathological changes in the kidney were analyzed through HE and PAS staining (bar = 20 *μ*m, *n* = 6-9 mice per group). (a) HE staining, (b) enlarged sections from (a), (c) representative sections of the thickening of glomerular basement membranes, and (d) representative sections of the tubular atrophy.

HE staining showed that the control group exhibited intact glomerular and tubular morphology, whereas the diabetic group exhibited glomerular hypertrophy, enlarged glomerular capsule, edema, and vacuolar degeneration of renal tubule accompanying with intense inflammatory cell infiltration. In contrast to the diabetic group, these histopathological alterations were ameliorated both in the LBP and MET groups (Figures 3(a) and 3(b)). The PAS staining results showed that comparing with the control group, the thickening of glomerular basement membranes and tubular atrophy was distributed throughout the analyzed sections in the diabetic group. These changes were eliminated after the administrations of LBP and MET, indicating that they have the potential ability to protect against diabetes-related kidney injury (Figures 3(c) and 3(d)).

### 3.4. The Anti-inflammatory Effects of LBP on Diabetic Mice

**Figure 4:**
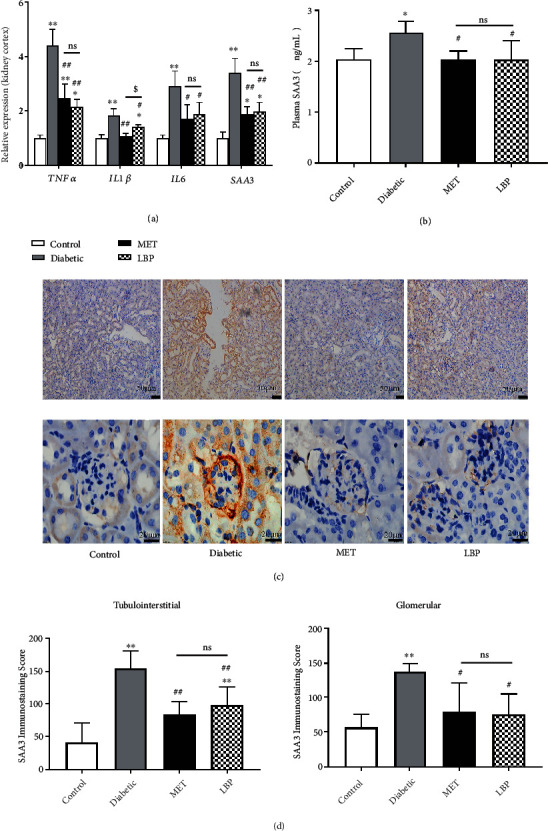
LBP inhibited the proinflammatory cytokine expression in diabetic mice: (a) the mRNA levels of TNF*α*, IL1b, IL6, and SAA3 in the kidney cortex; (b) the plasma level of SAA3; (c) the immunohistochemical analysis of SAA3 deposition in tubulointerstitium and glomerulus; (d) the quantification of SAA3 in the kidney by immunostaining scores. Data are expressed as the mean ± SD (*n* = 6-9 mice per group). ^∗^Control vs. other groups, ^∗^*p* < 0.05,  ^∗∗^*p* < 0.01; ^#^diabetic group vs. other groups, ^#^*p* < 0.05,  ^##^*p* < 0.01; ^$^MET vs. LBP, ^$^*p* < 0.05,  ^$$^*p* < 0.01; ns means no significance.

Hyperglycemia leads to kidney damage associated with the inflammation characterized by the release of multiple inflammatory factors [43]. In our study, hyperglycemic conditions resulted in significant upregulation of TNF*α*, IL1 *β*, IL6, and SAA3 gene expressions in the kidneys cortex as compared to the control (*p* < 0.01). The administration of LBP significantly decreased all of them and downregulated the elevated expressions of the abovementioned cytokines by 51.1%, 22.8%, 35.6%, and 42.4%, respectively (*p* < 0.05, Figure 4(a)). Similarly, as compared to the control group, the plasma concentration of SAA3 was remarkably elevated to 1.3-fold and its extensive protein depositions in the tubulointerstitium and glomerulus were also exhibited in the diabetic mice (*p* < 0.05). Conversely, those abnormal high levels were obviously reduced by 20.7%, 37.3%, and 45.6%, respectively, in the LBP-treated group (Figures 4(b)–4(d)).

### 3.5. LBP Inhibited Inflammation through Suppressing NF-*κ*B Activation in DN Mice

**Figure 5:**
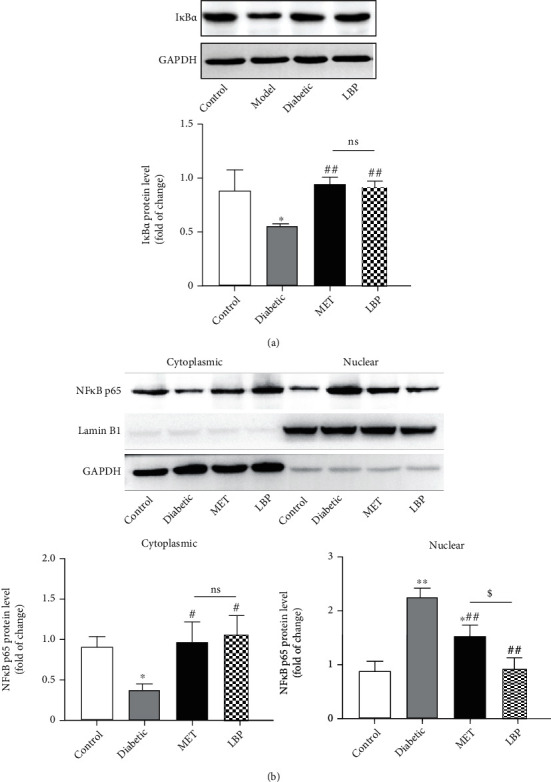
LBP inhibited NF-*κ*B signaling pathway in DN mice. (a) The protein level of I*κ*B*α* in the kidney and (b) the translocation of NF-*κ*B p65. Data are expressed as the mean ± SD (*n* = 6-9 mice per group). ^∗^Control vs. other groups, ^∗^*p* < 0.05,  ^∗∗^*p* < 0.01; ^#^diabetic vs. other groups, ^#^*p* < 0.05,  ^##^*p* < 0.01; ^$^MET vs. LBP, ^$^*p* < 0.05,  ^$$^*p* < 0.01; ns means no significance.

In order to determine whether the cytokines stimulated by hyperglycemia could activate the NF-*κ*B signaling pathway, the degradation of I*κ*B*α* and inhibition of p65 nuclear translocation in kidneys were analyzed by the western blot assay. As shown in Figure 5, the expression of I*κ*B*α* significantly decreased in the untreated diabetic mice, which was largely restored by LBP or MET treatment. Moreover, NF-*κ*B p65 expression was obviously increased in nuclear but decreased in cytoplasmic fraction in the diabetic group, suggesting that the nuclear translocation of NF-*κ*B p65 occurred. As expected, LBP, as well as MET, obviously reverted the nuclear translocation. Thus, the data suggested that LBP played the anti-inflammatory effect via inhibiting NF-*κ*B activation.

## 4. Discussion

Antidiabetic effects of LBP were studied extensively, and the capacity of LBP to influence blood glucose homeostasis has been reported in a number of literatures. These beneficial effects of LBP probably work through alleviating insulin resistance, increasing glucose utilization by peripheral tissues, inhibiting glucose uptake of intestines, and protecting pancreatic beta-cell proliferation [12, 17, 44]. Our data demonstrated that LBP obviously ameliorated glucose homeostasis and insulin resistance of the HFD/STZ-induced diabetic mice. Additionally, it exerted a renal protective effect by improving the renal function and histological features of DN. Moreover, it downregulated the levels of proinflammatory cytokines in the kidney via inhibiting NF-*κ*B nuclear activation. All these results suggest its therapeutic implication for preventing renal inflammation.

In previous studies, LBP reduced the levels of monocyte chemotactic protein 1 (MCP-1), intercellular cell adhesion molecule 1 (ICAM-1), IL2, IL6, TNF*α*, and interferon *α* (IFN*α*) by restraining the expression of NF-*κ*B p65 in the kidney cortex or inhibiting NF-*κ*B p65 phosphorylation in diabetic rabbit and rat models [45, 46]. Our study characterized NF-*κ*B p65 activation by its translocation from the cytoplasm into the nucleus in HFD/STZ-induced diabetic mice. NF-*κ*B contains a nuclear localization signal (NLS), which is masked by I*κ*B in unstimulated cells. Upon cell activation, I*κ*B is phosphorylated and degraded, and the NLS is unmasked, leading to the nuclear transport of NF-*κ*B. The translocation in the nucleus is thought to contribute to the inflammatory gene expressions [47, 48]. The levels of inflammatory factors such as IL2, IL6, TNF*α*, IFN*γ*, MCP-1, ICAM-1, vascular adhesion molecule 1 (VCAM-1), and toll-like receptor 4 (TLR-4) are reported to be downregulated by the inhibition of NF-*κ*B p65 nuclear translocation [23, 49]. In the present study, LBP significantly inhibited the translocation of NF-*κ*B p65 from the cytoplasm into the nucleus and reduced the circulating SAA3 and the expressions of TNF*α*, IL6, IL1 *β*, and SAA3 in the kidney of DN mice.

SAA plays a biologically mechanistic role in DN by triggering proinflammatory cascades through the activation of transcription factors such as NF-*κ*B [29]. Previous studies have reported that high circulating concentration and great amount of SAA in the kidney were found both in the patients with DN and the corresponding mouse models, and its protein deposition was present both in tubulointerstitium and glomerulus [29, 50]. In order to well elucidate the anti-inflammatory effect of LBP on DN, we highlighted its effect on SAA3. Based on our data, LBP decreased the elevated circulating SAA3 and reduced its protein deposition both in tubulointerstitium and glomerulus of DN mice. Future studies using human DN samples will further verify the correlation between SAA deposition and kidney injury.

It has been mentioned that LBP-1, LBP-2, LBP-3, LBP-4, and LBP-5 fractions were purified from the crude LBP, and the structure features were investigated in our recent study [51]. In order to find out which fraction of LBP is mainly responsible for preventing DN and the detailed mechanism of it, purified LBP will be used in future research, expecting to provide more scientific data on its pharmacological activity.

## 5. Conclusion

In conclusion, the evidence for the antidiabetic and renal protective effects of LBP was demonstrated in the present study. It significantly reduced the blood glucose level and ameliorated the insulin resistance of HFD/STZ-induced diabetic mice. Renal dysfunction and injury were also relieved by the supplement of LBP, as it was able to reduce the abnormally elevated serum creatinine, blood urea nitrogen, and urine microalbumin levels. Additionally, it ameliorated the histopathological changes of the renal glomeruli and tubules, which are associated with renal dysfunction and the biochemical indicators mentioned above. We also showed that LBP downregulated proinflammatory cytokines (TNF*α*, IL1 *β*, IL6, and SAA3) and inhibited NF-*κ*B p65 activation, which demonstrated its anti-inflammatory activity. Thus, our findings suggest that LBP can be used as an antidiabetic and anti-inflammatory agent to treat diabetic nephropathy.

## Figures and Tables

**Table 1:** Primer sequences of genes.

Name	Sequences (5′-3′)
*TNFα-F(mus)*	CCCTTTACTCTGACCCCTTTATTGT
*TNFα-R(mus)*	TGTCCCAGCATCTTGTGTTTCT
*IL1β-F(mus)*	TCCAGGATGAGGACATGAGCAC
*IL1β-R(mus)*	GAACGTCACACACCAGCAGGTTA
*IL6-F(mus)*	CCACTTCACAAGTCGGAGGCTTA
*IL6-R(mus)*	CCAGTTTGGTAGCATCCATCATTTC
*SAA3-F(mus)*	GACATGTGGCGAGCCTACTCTG
*SAA3-R(mus)*	CTCCATGTCCCGTGAACTTCTG
*β-Actin-F(mus)*	GTGCTATGTTGCTCTAGACTTCG
*β-Actin-R(mus)*	ATGCCACAGGATTCCATACC

## Data Availability

The data used to support the findings of this study are available from the corresponding author upon request.

## Abbreviations

LBP: *Lycium barbarum* polysaccharides
DN: Diabetic nephropathy
HOMA-IR: Homeostasis model assessment-insulin resistance
sCr: Serum creatinine
BUN: Blood urea nitrogen
HE: Hematoxylin and eosin
PAS: Periodic acid-Schiff
SAA3: Serum amyloid A3
TNF*α*: Tumor necrosis factor *α*
IL1 *β*: Interleukin 1 *β*
IL6: Interleukin 6.

## Supplementary Materials

Supplementary Materials Figure S1: HPLC chromatograms of monosaccharide composition of *Lycium barbarum* polysaccharide (LBP) (1: mannose, 2: rhamnose, 3: galacturonic acid, 4: glucose, 5: galactose, and 6: arabinose). PMP (1-phenyl-3-methyl-5-pyrazolone) derivatives of six standard monosaccharides (a) and component monosaccharide in LBP (b). Figure S2: dose-response of metformin on the blood glucose of diabetic mice. (a) fasting blood glucose level after 4 weeks of treatment; (b) random blood glucose level after 4 weeks of treatment. Data represent the mean ± SD (*n* = 6-9 mice per group). ^∗^Control vs. other groups, ^∗^*p* < 0.05,  ^∗∗^*p* < 0.01; ^#^diabetic vs. other groups, ^#^*p* < 0.05,  ^##^*p* < 0.01; ^$^MET (400 mg/kg body weight) vs. MET (100, 200 mg/kg body weight), ^$^*p* < 0.05,  ^$$^*p* < 0.01. Figure S3: the schematic representation of the experimental procedure. Table S1: the formulation of the high-fat diet.
