# Supplementary material for: Effect of Lycium barbarum Polysaccharide on Decreasing Serum Amyloid A3 Expression through Inhibiting NF-κB Activation in a Mouse Model of Diabetic Nephropathy
Source: Anal Cell Pathol (Amst). 2022 Jan 29;2022:7847135. doi: 10.1155/2022/7847135 (PMC8817866; doi:10.1155/2022/7847135)
Supplement: Supplementary Materials — Figure S1: HPLC chromatograms of monosaccharide composition of Lycium barbarum polysaccharide (LBP) (1: mannose, 2: rhamnose, 3: galacturonic acid, 4: glucose, 5: galactose, and 6: arabinose). PMP (1-phenyl-3-methyl-5-pyrazolone) derivatives of six standard monosaccharides (a) and component monosaccharide in LBP (b). Figure S2: dose-response of metformin on the blood glucose of diabetic mice. (a) fasting blood glucose level after 4 weeks of treatment; (b) random blood glucose level after 4 weeks of treatment. Data represent the mean ± SD (n = 6-9 mice per group). ∗Control vs. other groups, ∗p < 0.05, ∗∗p < 0.01; #diabetic vs. other groups, #p < 0.05, ##p < 0.01; $MET (400 mg/kg body weight) vs. MET (100, 200 mg/kg body weight), $p < 0.05, $$p < 0.01. Figure S3: the schematic representation of the experimental procedure. Table S1: the formulation of the high-fat diet. [file 7847135.f1.docx]

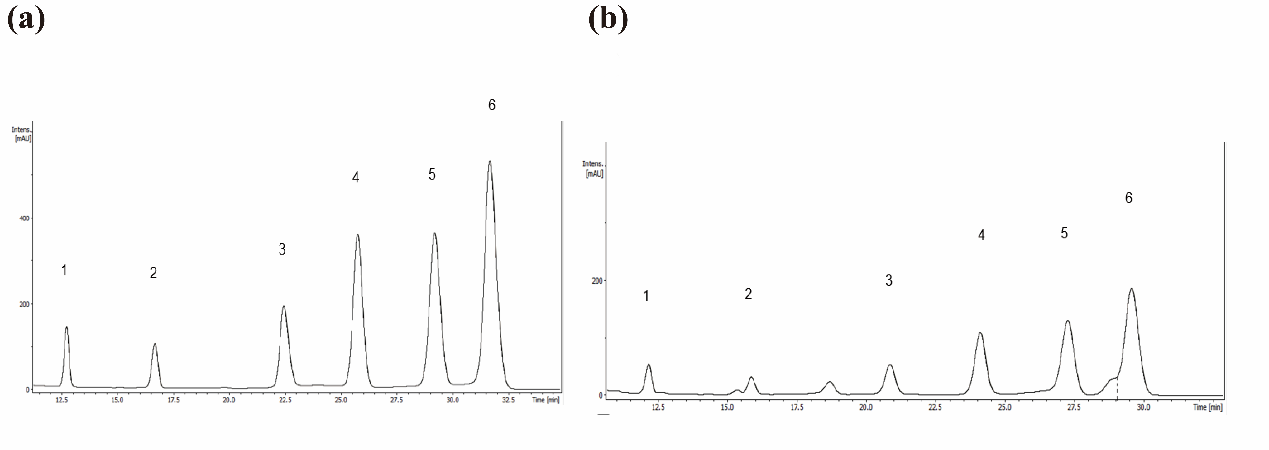
**Supplementary Materials**

**Figure S1. HPLC chromatograms of monosaccharide composition of *Lycium barbarum* polysaccharide (LBP)** (1-mannose, 2-rhamnose, 3-galacturonic acid, 4-glucose, 5-galactose, 6-arabinose)**.** PMP (1-Phenyl-3-methyl-5-pyrazolone) derivatives of six standard monosaccharide (a) and component monosaccharide in LBP (b).


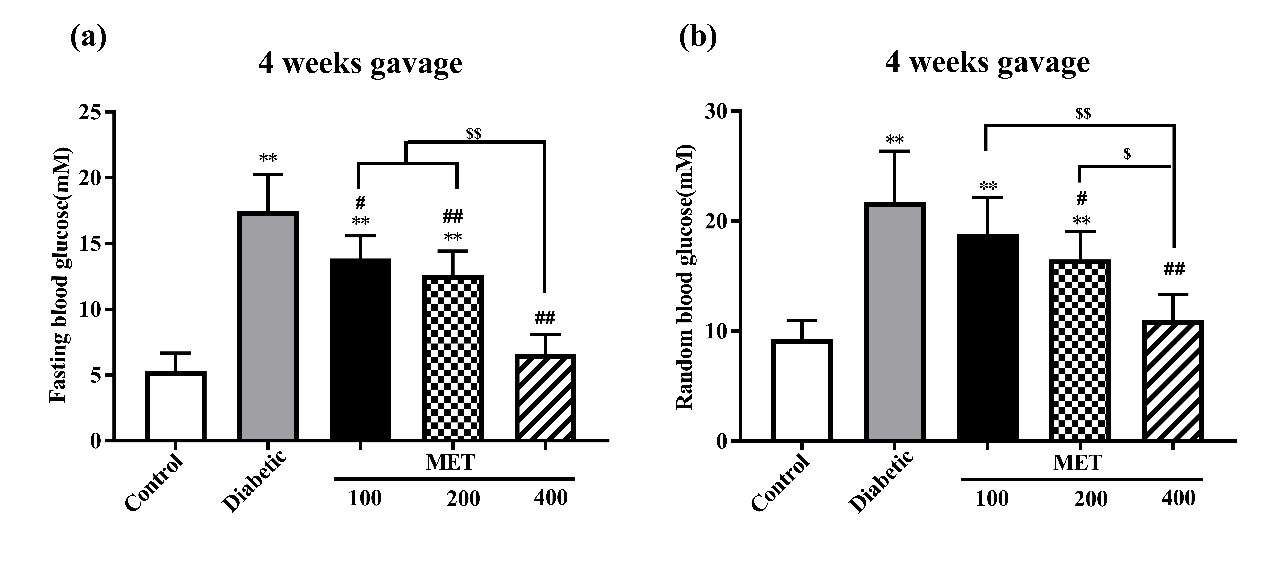


**Figure S2. Dose-response of metformin on the blood glucose of diabetic mice.** (a) fasting blood glucose level after 4 weeks treatment, (b) random blood glucose level after 4 weeks treatment. Data represent mean ± SD (n=6-9 mice per group). **^*^**Control vs other groups, ^*^*p* ＜ 0.05, ^**^*p* ＜ 0.01; **^#^**diabetic vs. other groups, ^#^*p* ＜ 0.05, ^##^*p* ＜ 0.01; **^$^**MET (400mg/kg bodyweight) vs. MET (100, 200mg/kg bodyweight), ^$^*p* ＜ 0.05, ^$$^*p* ＜ 0.01.


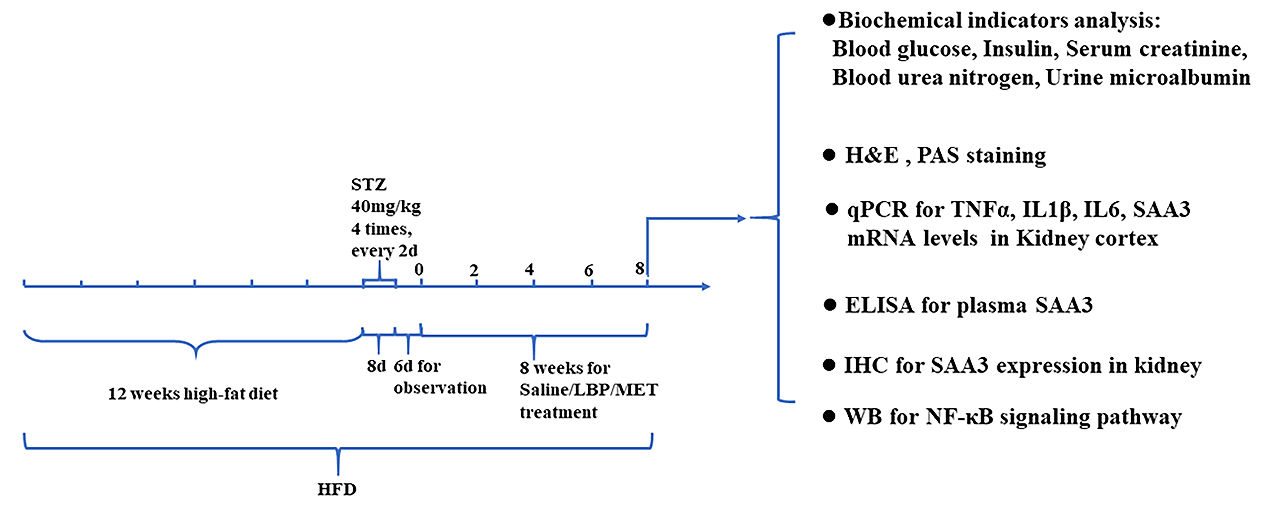


**Figure S3 the schematic representation of the experimental procedure.**

**Table S1 The formulation of the high-fat diet**

| Class description | Ingredients | Grams (g) |
| --- | --- | --- |
| Protein | Casein, Lactic | 200.00 |
| Protein | Cystine, L | 3.00 |
| Carbohydrate | Lodex 10 | 125.00 |
| Carbohydrate | Sucrose | 72.80 |
| Fiber | Solka Floc, FCC200 | 50.00 |
| Fat | Lard | 245.00 |
| Fat | Soybean Oil | 25.00 |
| Mineral | S10026B (Mineral Mix) | 50.00 |
| Vitamin | Choline Bitartrate | 2.00 |
| Vitamin | V10001C (Vitamin Mix) | 1.00 |
| Dye | Blue | 0.05 |
| Total |  | 773.85 |

Note: Research Diets, cat No. D12492 (60 kcal% fat).
